# Evolution of ubiquitin, cytoskeleton, and vesicular trafficking machinery in giant viruses

**DOI:** 10.1128/jvi.01715-24

**Published:** 2025-02-11

**Authors:** Sangita Karki, Frank O. Aylward

**Affiliations:** 1 Department of Biological Sciences, Virginia Tech 309993 , Blacksburg, Virginia, USA; 2 Center for Emerging, Zoonotic, and Arthropod-borne Pathogens, Virginia Tech 1757 , Blacksburg, Virginia, USA; Michigan State University, East Lansing, Michigan, USA

**Keywords:** giant viruses, *Nucleocytoviricota*, eukaryotic signature proteins, viral origins, viral diversity

## Abstract

Members of the phylum *Nucleocytoviricota*, which include “giant viruses” known for their large physical dimensions and genome lengths, are a diverse group of dsDNA viruses that infect a wide range of eukaryotic hosts. The genomes of nucleocytoviruses frequently encode eukaryotic signature proteins (ESPs) such as RNA- and DNA-processing proteins, vesicular trafficking factors, cytoskeletal components, and proteins involved in ubiquitin signaling. Despite the prevalence of these genes in many nucleocytoviruses, the timing and number of gene acquisitions remains unclear. While the presence of DNA- and RNA-processing proteins in nucleocytoviruses likely reflects ancient gene transfers, the origins and evolutionary history of other proteins are largely unknown. In this study, we examined the distribution and evolutionary history of a subset of viral-encoded ESPs (vESPs) that are widespread in nucleocytoviruses. Our results demonstrate that most vESPs involved in vesicular trafficking were acquired multiple times independently by nucleocytoviruses at different time points after the emergence of the eukaryotic supergroups, while viral proteins associated with cytoskeletal and ubiquitin system proteins exhibited a more complex evolutionary pattern exhibited by both shallow and deep branching viral clades. This pattern reveals a dynamic interplay between the co-evoluton of eukaryotes and their viruses, suggesting that the viral acquisition of many genes involved in cellular processes has occurred both through ancient and more recent horizontal gene transfers. The timing and frequency of these gene acquisitions may provide insight into their role and functional significance during viral infection.

**IMPORTANCE:**

This research is pertinent for understanding the evolution of nucleocytoviruses and their interactions with eukaryotic hosts. By investigating the distribution and evolutionary history of viral-encoded eukaryotic signature proteins, the study reveals gene transfer patterns, highlighting how viruses acquire genes that allow them to manipulate host cellular processes. Identifying the timing and frequency of gene acquisitions related to essential cellular functions provides insights into their roles during viral infections. This work expands our understanding of viral diversity and adaptability, contributing valuable knowledge to virology and evolutionary biology, while offering new perspectives on the relationship between viruses and their hosts.

## INTRODUCTION

The discovery of giant viruses has dramatically expanded our understanding of the limits of viral size and genome complexity. These viruses belong to the phylum *Nucleocytoviricota*, which comprises six orders, 11 families, and an expanding number of family-level lineages (1). Although they are found in diverse environments, they are particularly abundant in the oceans (2–6) and infect a wide range of hosts, ranging from the smallest known free-living eukaryote (7) to metazoans (8). Members of families such as *Poxviridae*, *Asfarviridae*, and *Iridoviridae* include well-known vertebrate pathogens (9–11) while members of the *Mimiviridae, Phycodnaviridae,* and *Marseilleviridae* primarily infect protists and algae (12–14). Phylogenomic studies across these groups have provided evidence that they have a shared evolutionary origin and likely evolved from a smaller viral ancestor (15–17). Gene acquisition from their hosts coupled with gene duplication likely contributed to the subsequent expansion of genome size and complexity in this lineage (15, 18–20).

A common theme in recent years has been the discovery of numerous “cell-like” genes in nucleocytoviruses that are common in cellular life but are rare or absent from other viral lineages (21). These include genes involved in translation (22, 23), transcription (24), DNA replication and repair pathways (25–27), diverse metabolic pathways including the TCA cycle and glycolysis (28), cytoskeletal structure (29–31), membrane trafficking (32), and ubiquitin (Ub) signaling (33). In some cases, viruses have acquired these genes from their hosts recently, and viral homologs form well-defined clades within broader eukaryotic lineages. This appears to be the case for ammonia transporters, some rhodopsins, and sphingolipid metabolism genes (34–36). In other cases, such as histones, DNA polymerase subunits, and some tRNA synthetases, viral genes are the result of ancient gene transfer events that may have occurred prior to the emergence of the last eukaryotic common ancestor (LECA), and their evolutionary relationship to cellular homologs is less clear (37–40). Recent studies have shown that nucleocytoviruses often endogenize their genomes into those of their host (41, 42) indicating that virus-to-host gene transfer may also be a possibility in some cases. Phylogenetic trees of multi-subunit RNA polymerase (24), DNA topoisomerase IIA (43), actin (31), and DNA polymerase delta (38) are examples of ancient gene transfers that have been proposed, although the direction of these transfer events is difficult to definitively ascertain. Regardless of the direction of gene transfer, these studies have begun to elucidate ancient patterns of gene transfer between viruses and eukaryotes that underscore the long co-evolutionary history of eukaryotes and nucleocytoviruses.

The origin of eukaryotes and their viruses represents a major transition in the evolution of life on Earth (44), and yet the details of this process remain a riddle (45). Because nucleocytoviruses belong to an ancient lineage that potentially predates LECA, it is possible that examination of eukaryote-virus gene exchange may provide important details into the early evolution of both lineages (46). Interestingly, many nucleocytoviruses encode eukaryotic signature proteins (ESPs) that are involved in central processes in eukaryotic cells, such as DNA and RNA processing, vesicular trafficking, cytoskeletal structure, and ubiquitin signaling. ESPs are common in eukaryotes but typically rare or absent in bacteria and archaea, although an increasing number have recently been discovered in Asgard archaea (47), and examination of their evolution therefore can provide key clues into the emergence of eukaryotes (48, 49). In this study, we focus on viral eukaryotic signal proteins (vESPs) associated with ubiquitin signaling, vesicular trafficking, and cytoskeletal dynamics, which were chosen because of their prevalence in some nucleocytovirus lineages as well as their typical absence in cellular groups other than eukaryotes. By performing a detailed phylogenetic analysis of these proteins, we aim to uncover patterns of gene acquisition and evolutionary history that reflect the complex interplay between nucleocytoviruses and their eukaryotic hosts. Our findings provide insights into the ancient co-evolutionary dynamics of nucleocytoviruses and their hosts.

## RESULTS

### Occurrence of vESPs

**Fig 1:**
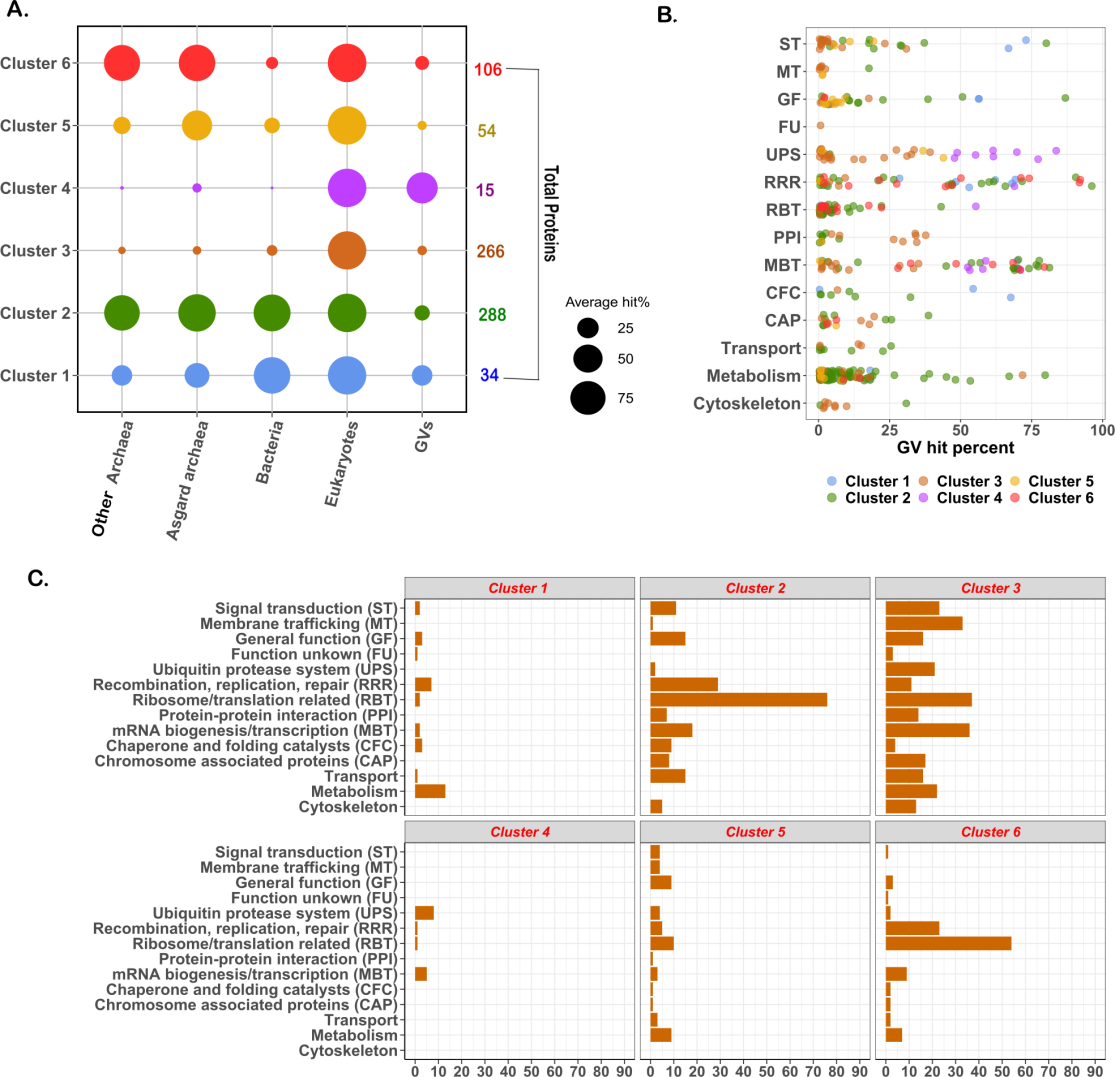
Clustering and functional annotation of ESPs. (**A**) Unsupervised clustering of 763 eukaryotic proteins (ESPs) revealed six different clusters based on their prevalence in different groups. Out of 763, 500 ESPs were present in at least one nucleocytovirus genome. (**B**) Functional categories of protein domains present in greater than five nucleocytovirus genomes irrespective of clusters. (**C**) Functional annotation of protein domains belonging to different clusters. (Abbreviation: ST, signal transduction; MT, membrane trafficking; GF, general function; UPS, ubiquitin-protease system; RRR, replication, repair, and recombination; RBT, ribosome/translation related; PPI, protein-protein interaction; CFC, chaperone and folding catalysts; MBT, mRNA biogenesis and transcription; CAP, chromosome associated proteins; FU, function unknown).

We first sought to identify a set of protein families that are commonly found in both eukaryotes and nucleocytoviruses. We identified a set of 763 Pfam domains that are present in greater than 95% of the eukaryotic genomes surveyed, and 500 of these could be identified in at least one nucleocytovirus genome. These Pfams could be clustered into six groups depending on their occurrence across different lineages (Table S1). Clusters 1 and 2 are broadly distributed across all cellular lineages (pan-cellular groups), clusters 3 and 4 are found primarily in eukaryotes (true vESPs), and clusters 5 and 6 are found primarily in eukaryotes and archaea (archaea-vESPs) (Fig. 1A). Many vESPs were only found in a small number of viral genomes and are not suitable for phylogenetic reconstruction; we therefore examined the prevalence of vESPs from different clusters across the *Nucleocytoviricota* to identify those that are broadly represented in this viral phylum (Fig. 1B and C).

**Fig 2:**
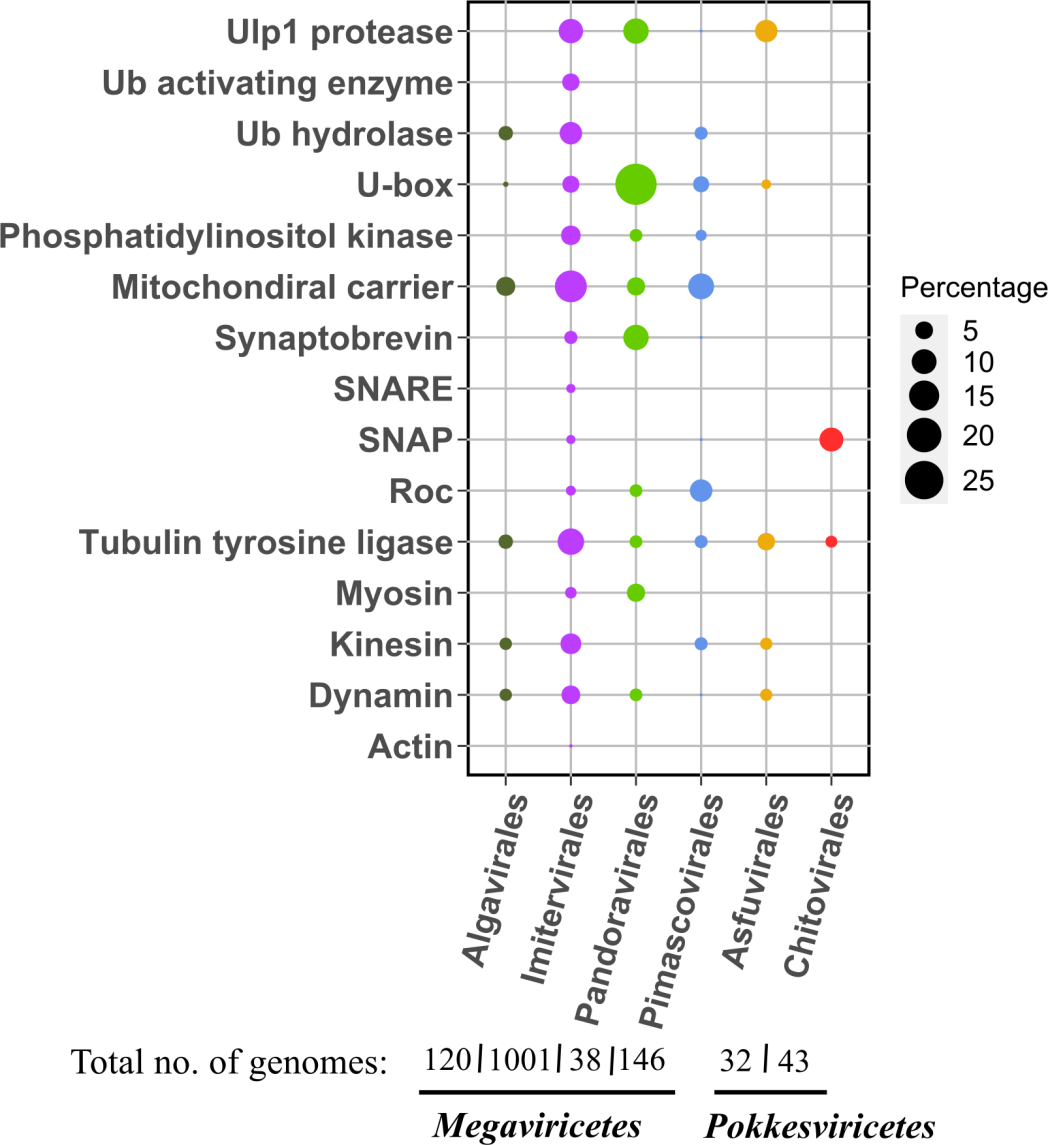
Prevalence of selected cytoskeleton, membrane trafficking and transport, and ubiquitin-protease system proteins in the nucleocytoviruses. The bubble represents the percentage of the protein encoded by the genomes within the orders. Percentages are based on 1,001 total *Imitervirales*, 146 *Pimascovirales*, 120 *Algavirales*, 43 *Chitovirales*, 38 Pandoravirales, and 32 *Asfuvirales* in the Giant Virus database.

**Fig 3:**
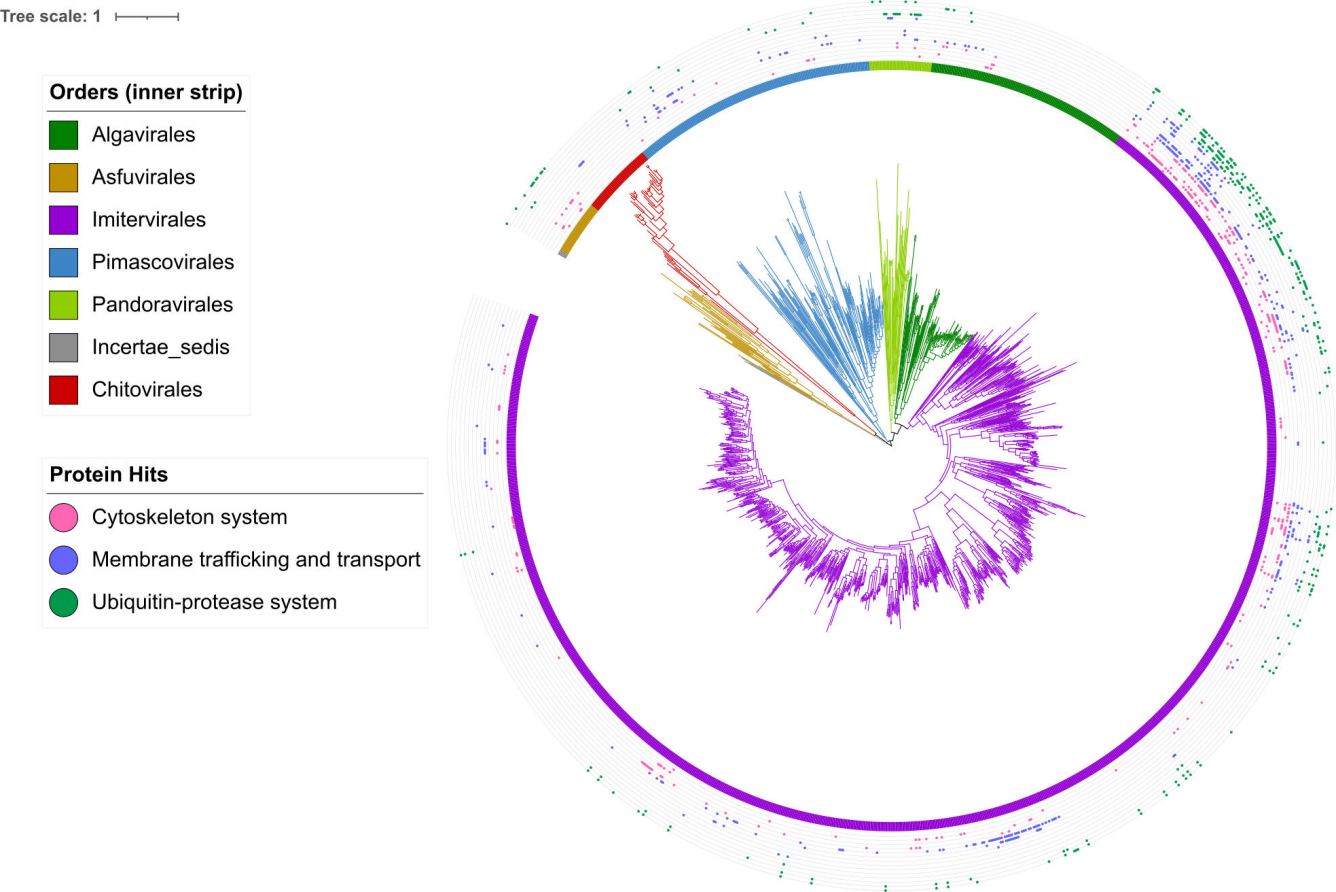
Phylogeny of the *Nucleocytoviricota* with vESP distributions mapped. The inner strip represents nucleocytoviruses order while outer dots represent cytoskeleton system, membrane trafficking, and ubiquitin-protease system protein hits to the nucleocytoviruses orders.

To identify ESPs that are prevalent in nucleocytoviruses and eukaryotes but largely absent elsewhere, we searched Pfams that were represented in greater than 10 nucleocytovirus genomes and were mostly prevalent in clusters 3 and 4, because these clusters contained proteins that were mostly unique to eukaryotes and nucleocytoviruses. A preponderance of these proteins is involved in vesicular trafficking, ubiquitin signaling, cytoskeletal structure, mRNA biogenesis, and DNA processing and replication. Because several phylogenetic analyses of eukaryotic and viral genes involved in RNA and DNA processing have already been reported and revealed ancient evolutionary links between eukaryotes and nucleocytoviruses (24, 37–39, 43), we focused our analysis on protein families involved in membrane trafficking and transport, the cytoskeletal system, and ubiquitin-protease system. For proteins involved in the cytoskeletal system, we chose actin, myosin, kinesin, dynamin, and tubulin tyrosine ligase (TTL); for those involved in membrane trafficking, we chose phosphatidylinositol kinase (PIK), soluble N-ethylmaleimide-sensitive factor attachment protein (SNAP), soluble N-ethylmaleimide-sensitive factor activating protein receptor (SNARE), ras of complex (Roc), synaptobrevin, and the mitochondrial carrier protein; and for genes involved in the ubiquitin-protease system, we chose Ulp1 protease, U-box domain, ubiquitin hydrolase, and the ubiquitin-activating enzyme (UBA) (Fig. 2). These proteins were collectively found in a wide range of nucleocytoviruses (Fig. 2), and vESPs had amino-acid identity to eukaryotic homologs that ranged from 20 to 74% (details in Table S3). We also mapped these vESPs to the *Nucleocytoviricota* orders (Fig. 3). The genomes within the *Megaviricetes* (and in particular the *Imitervirales*) contain the most vESPs, likely due to the larger and more complex genomes in this group (Fig. 3).

Given the divergent nature of many vESPs that we examined here, we used several quality-control filters to examine the reliability of the trees that we generated. In order to evaluate the phylogenetic strength of the vESP trees we constructed, we calculated the tree certainty (TC) metric for each tree. This metric varies between zero and one, with higher values indicating fewer contrasting bipartitions and a more reliable topology (50). Longer genes with a stronger phylogenetic signal typically exhibit larger TC values (51), and here we report only trees with TC >0.5. It is important to note, however, that TC values are influenced by factors such as the size of the tree, the global support values, and the type of bootstrap replicates used. In this study, we used ultrafast bootstrap (“UFBoot”) replicates. All TC scores are provided for each tree and are also included in Table S2. Similarly, the UF bootstrap support values are also depicted in supplementary figures, Fig. S4 to S6.

### Evolution of vESPs involved in cytoskeletal dynamics

**Fig 4:**
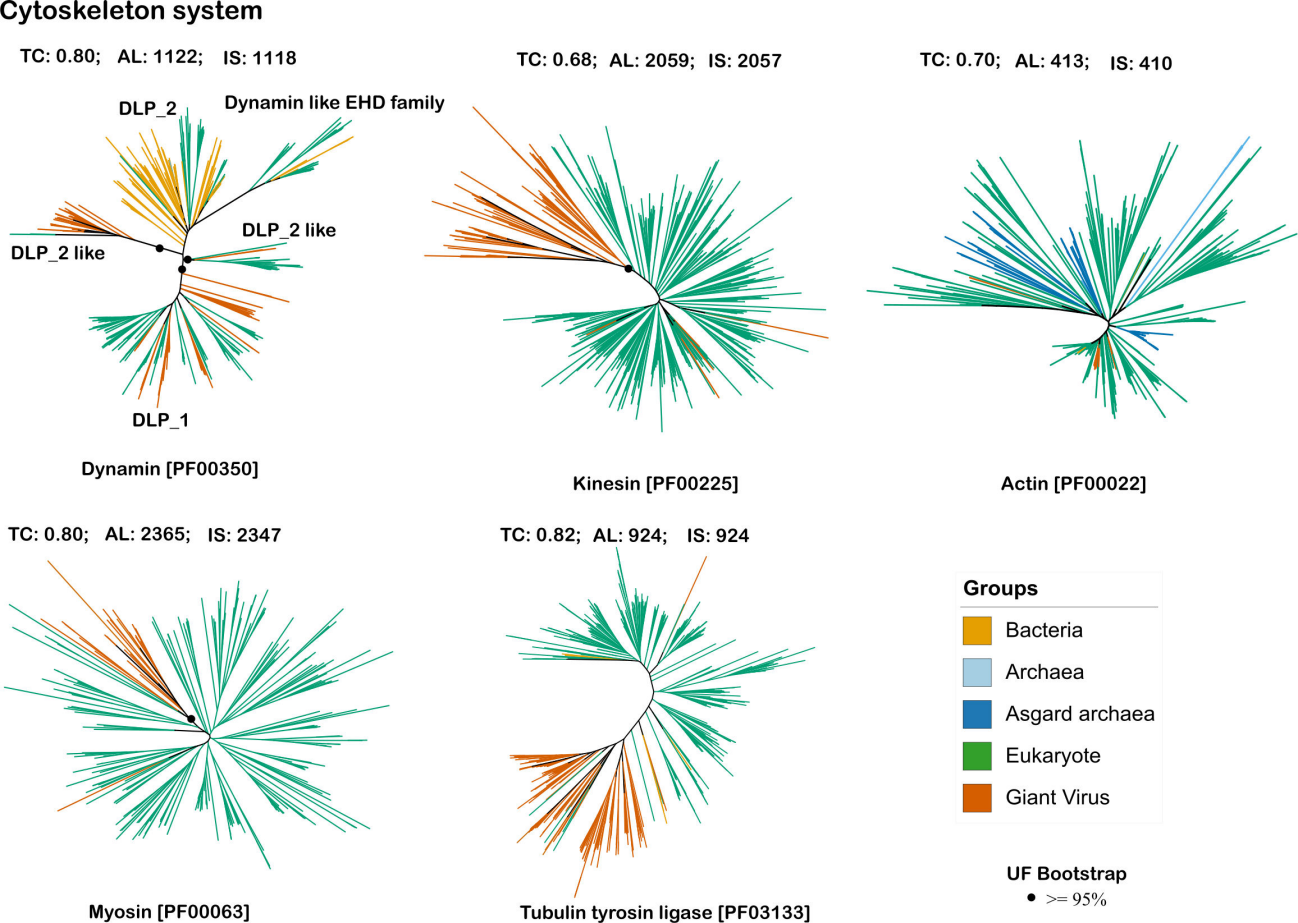
Phylogenetic trees for vESPs involved in cytoskeletal dynamics.TC refers to the tree certainty value for the given tree, AL refers to alignment length, and IS refers to the number of parsimony informative sites. The dots represent ultrafast bootstrap support values greater than 95%, only for the major viral branches. All bootstrap support values are provided in supplementary figures, Fig. S4.

Our analysis showed that all the cytoskeleton protein domains were encoded by the members of *Imitervirales*. Actin homologs could only be found in members of the *Imitervirales*, while myosin homologs were restricted to the *Imitervirales* and the proposed order Pandoravirales. Kinesin, dynamin, and tubulin tyrosine ligase homologs were found in a broader range of orders (Fig. 2). In our phylogenetic analysis, it was notable that the breadth of the viral clades varies between trees for the cytoskeleton proteins. Deep viral clades were evident for the myosin, kinesin, and tyrosine tubulin ligase protein families, suggesting that these proteins were acquired by nucleocytoviruses only a small number of times throughout their diversification (Fig. 4; Fig. S1). We hypothesize that proteins belonging to these large viral clades are more likely to have conserved roles in infection across diverse viruses. By contrast, viral dynamin homologs are distributed into several distinct clades, indicating that these were acquired multiple times independently. Due to these independent origins, it is possible that different clades of viral dynamin have distinct roles during infection.

Viral homologs to cytoskeletal proteins may play a range of different roles in manipulating host physiology during infection. Past research has shown that African swine fever virus (ASFV), the most well-studied member of *Asfuvirales,* uses conventional kinesin and microtubules to transport viral particles from perinuclear assembly sites to the cell surface. This work showed that virus movement to the cell periphery was inhibited when microtubules were depolymerized (50). TTL stabilizes microtubules by adding a tyrosine residue to α-tubulin, which regulates microtubule dynamics and supports essential cellular processes (51). It is therefore possible that viral-encoded tubulin tyrosine ligase plays a role in viral trafficking and egress during infection. Viral-encoded kinesin, actin, and myosin may play similar roles.

Viral homologs of dynamin were also present in nucleocytoviruses (Fig. 4; Fig. S1). The dynamin superfamily comprises numerous members that are essential to membrane remodeling processes, such as the fission and fusion of intracellular vesicles and organelles like mitochondria and chloroplasts, as well as cytokinesis. Certain members, like the MX proteins, inhibit a wide range of viruses by blocking an early stage of the replication cycle (52). Due to their significance, the classification of the dynamin superfamily continues to be developed. Broadly, it includes classical dynamins and dynamin-related proteins, which encompass a diverse range of dynamin homologs (53). In our phylogenetic tree (Fig. 4), nucleocytoviruses are distributed across multiple clades. One major viral clade is positioned near the DLP_2 sequences, which includes classical dynamins, mitofusins, guanylate-binding proteins, and bacterial DLPs (54), suggesting a potential functionality related to DLP_2 groups. Conversely, multiple independent viral acquisitions were evident for nucleocytoviruses clustering with eukaryotic DLP_1, a group that includes classical dynamins, dynamin-like proteins, and MX proteins, which contribute to host viral resistance (54), indicating that the nucleocytoviruses might have acquired these for counteracting the viral resistance by the host cell. These findings highlight the evolutionary complexity of the dynamin superfamily. A recent study provided a detailed phylogenetic analysis of dynamin-like proteins and provided experimental evidence that members of the DLP_1 group were involved in mitochondrial remodeling during infection (55). The presence of these proteins in viral genomes is significant because it indicates these viruses may have a more direct level of control over these processes than they would if they relied on manipulating host-encoded proteins.

A study by Kijima et al. (29) suggested that nucleocytoviruses acquired myosin homologs through gene transfers from the SAR (stramenopiles, alveolates, and rhizarians) supergroups of eukaryotes. Research studying the transcriptional activity of giant viruses in coastal marine systems also identified viral transcripts of cytoskeleton proteins like kinesin and myosin. Both of these studies indicated the existence of deep-branching viral clades nested within eukaryotic clades as well as a few more recent viral acquisitions (30). Deep-branching viral clades for viral actin genes were shown by Da Cunha and colleagues, who also presented a possible evolutionary scenario in which eukaryotic actin was originally acquired from viruses (31). Cytoskeletal proteins are hypothesized to play a critical role in the manipulation of host cytoskeletal dynamics, which is essential for viral cell entry and egress (56). Therefore, these proteins may significantly influence the interactions between viruses and their hosts (57). The prevalence of these proteins in deep-branching viral lineages suggests that their relatively older acquisition is related to their fundamental role in viral entry and exit processes.

### Evolution of vESPs involved in membrane trafficking and transport

**Fig 5:**
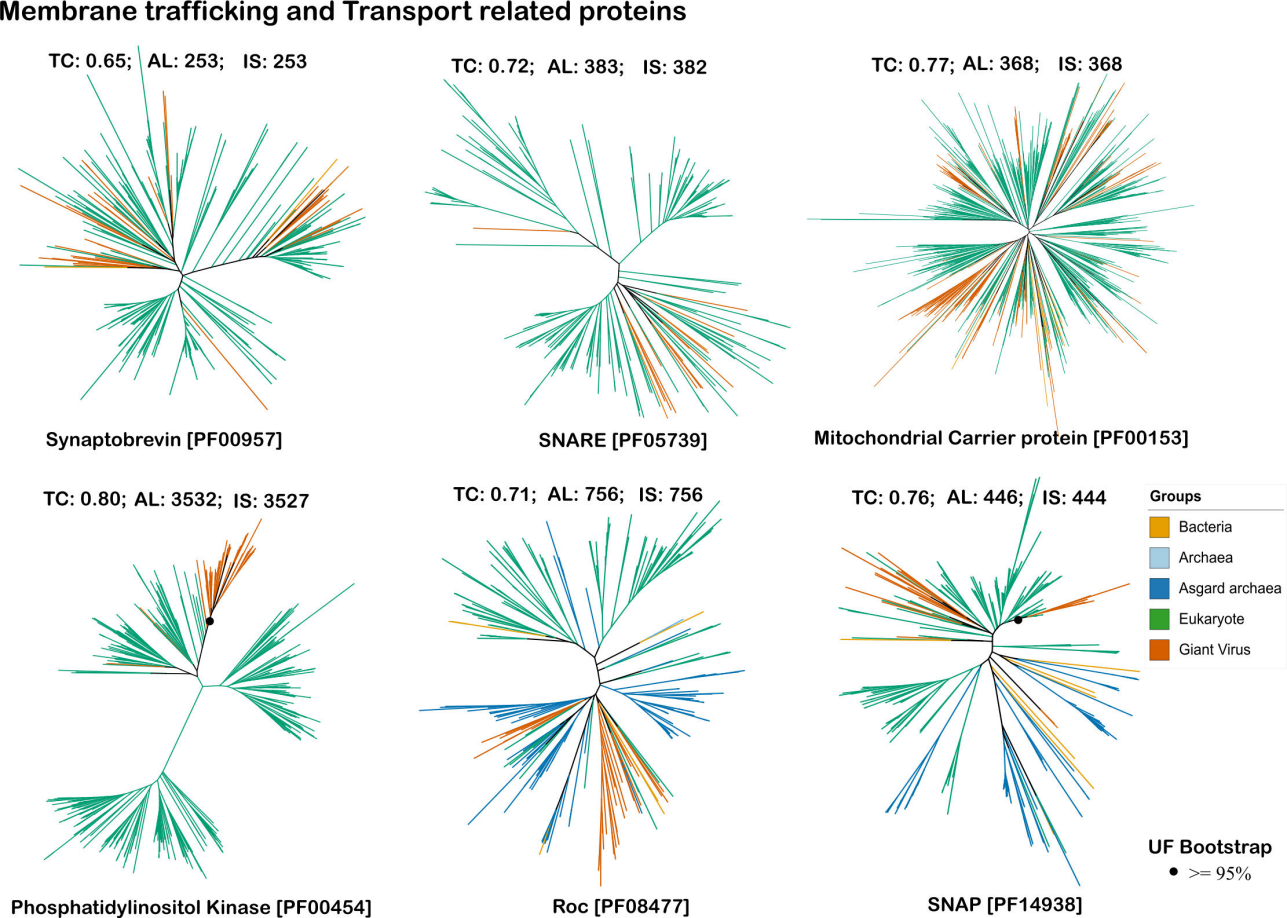
Phylogenetic trees for vESPs involved in membrane trafficking and transport system.TC refers to the tree certainty value for the given tree, AL refers to alignment length, and IS refers to the number of parsimony informative sites. The dots represent ultrafast bootstrap support values greater than 95%, only for the major viral branches. All bootstrap support values are provided in supplementary figures, Fig. S5.

All membrane trafficking, signal transduction, and transport proteins were encoded by members of *Imitervirales*. Synaptobrevin, Roc, and PIK also had hits to proteins found in genomes within the Pandoravirales and *Pimascovirales,* while the mitochondrial carrier protein also had hits to proteins found in genomes within the Pandoravirales*, Pimascovirales,* and *Algavirales*. SNAP was also found in genomes within the *Pimascovirales* and *Chitovirales* (Fig. 2). We found that the membrane trafficking and transport proteins such as the SNARE, synaptobrevin, mitochondrial carrier protein, and SNAP vESPs are scattered broadly among eukaryotic homologs, revealing multiple independent acquisitions at various time points. Signal transducing proteins such as the phosphatidylinositol kinase and Roc had deep large clades with few instances of multiple acquisitions (Fig. 5; Fig. S2).

Interestingly, for SNAP, homologs found in the *Pokkesviricetes* (specifically, *Chitovirales*) clustered within a clade of multicellular eukaryotic hosts, suggesting that viruses acquired this specific protein from the hosts it infects. The *Megaviricetes* and *Pokkesviricetes* did not cluster together in this tree, indicating that the acquisition of SNAP proteins occurred independently in these lineages (see Supplementary figures, Fig. S2). Some experimental work has been done to examine the role of these viral-encoded proteins during infection; a past study has shown that the fowlpox virus encodes a homolog of mammalian SNAP that is crucial to vesicular transport in the exocytic pathway (58).

Our results also show that distinct nucleocytoviruses encode their own Roc (Ras/GTPase domain) proteins (Fig. 5). This may stem from the unique biology and large genomes of *Nucleocytoviricota*, which could require the use of a specialized Roc domain for manipulating critical host cellular processes like signal transduction and cell growth regulation during infection. The presence of this signal transduction domain, acquired by different groups within *Megaviricetes*, suggests an evolutionary adaptation that allows these viruses to control their host’s cellular environment more directly. Rab GTPase, a protein involved in cellular membrane trafficking that belongs to the Ras/GTPase superfamily, was reported in mimivirus, and a role in the formation of the virion membrane during its assembly process was suggested (59). Another study has shown that herpesviruses utilize cellular signaling molecules like the Ras signaling pathway to enhance their replication, many of which are also involved in critical cellular functions like proliferation, differentiation, and cell survival (60). Herpesviruses belong to a distinct lineage of large DNA viruses that would have acquired this protein convergently, supporting the view of convergence across distinct viruses.

Our phylogenetic analysis showed that several members of the *Megaviricetes* encode the mitochondrial carrier protein, and that this protein was likely acquired multiple times independently by viruses (Fig. 5; Fig. S2). The significance of this acquisition was discussed in a past study, where it was initially reported that mimivirus encodes a mitochondrial transport protein known as VMC1 (viral mitochondrial carrier) (61). Based on the transport assays carried out with the expressed VMC1 protein in lactococcal membranes, it was suggested that the VMC1 facilitates the transport of various nucleotide triphosphates (61) and this transport process enables mimivirus to utilize mitochondrial nucleotide pools and support the replication of its genome (61, 62).

Our findings are consistent with a phylogenetic study where a highly divergent mimivirus Rab GTPase, a crucial regulator of membrane trafficking in eukaryotes, was reported to be acquired from unicellular eukaryotes (59). Moreover, a recent study that confirmed the activity of some viral vesicular trafficking components also suggested that these proteins had been acquired multiple times by viruses (32). This study suggested that many of these proteins may serve to interfere with normal host processes during infection and thereby facilitate viral takeover of the cell. Moreover, vesicular trafficking plays an important role in virion morphogenesis by recruiting membranes derived from the endoplasmic reticulum to the virus factory, where they contribute to the formation of new virions (63). In addition, it has also been suggested that membrane vesicles are crucial for the processes of cell entry and egress in giant viruses (64). These insights advance our understanding of how viruses may harness and repurpose host cellular machinery to facilitate their infection cycle, including entry, replication, and egress.

### Evolution of vESPs involved in ubiquitin-protease system

In our analysis, ubiquitin-protease proteins were predominately found in genomes within the *Imitervirales*. In addition, Ulp1 protease also had hits to proteins found in genomes within the *Asfuvirales* and Pandoravirales, Ub hydrolase was also encoded by genomes in the *Algavirales* and *Pimascovirales,* while the U-box domain had hits to genomes in the *Asfuvirales,* Pandoravirales*, Algavirales*, and *Pimascovirales* (Fig. 2). Interestingly, for the Ulp1 protease, the *Asfuvirales* (class *Pokkesviricetes*) were clustering within the other *Megaviricetes,* suggesting that inter-viral gene transfer may be responsible for the distribution of this gene in extant viruses (see supplementary figures, Fig. S3).

**Fig 6:**
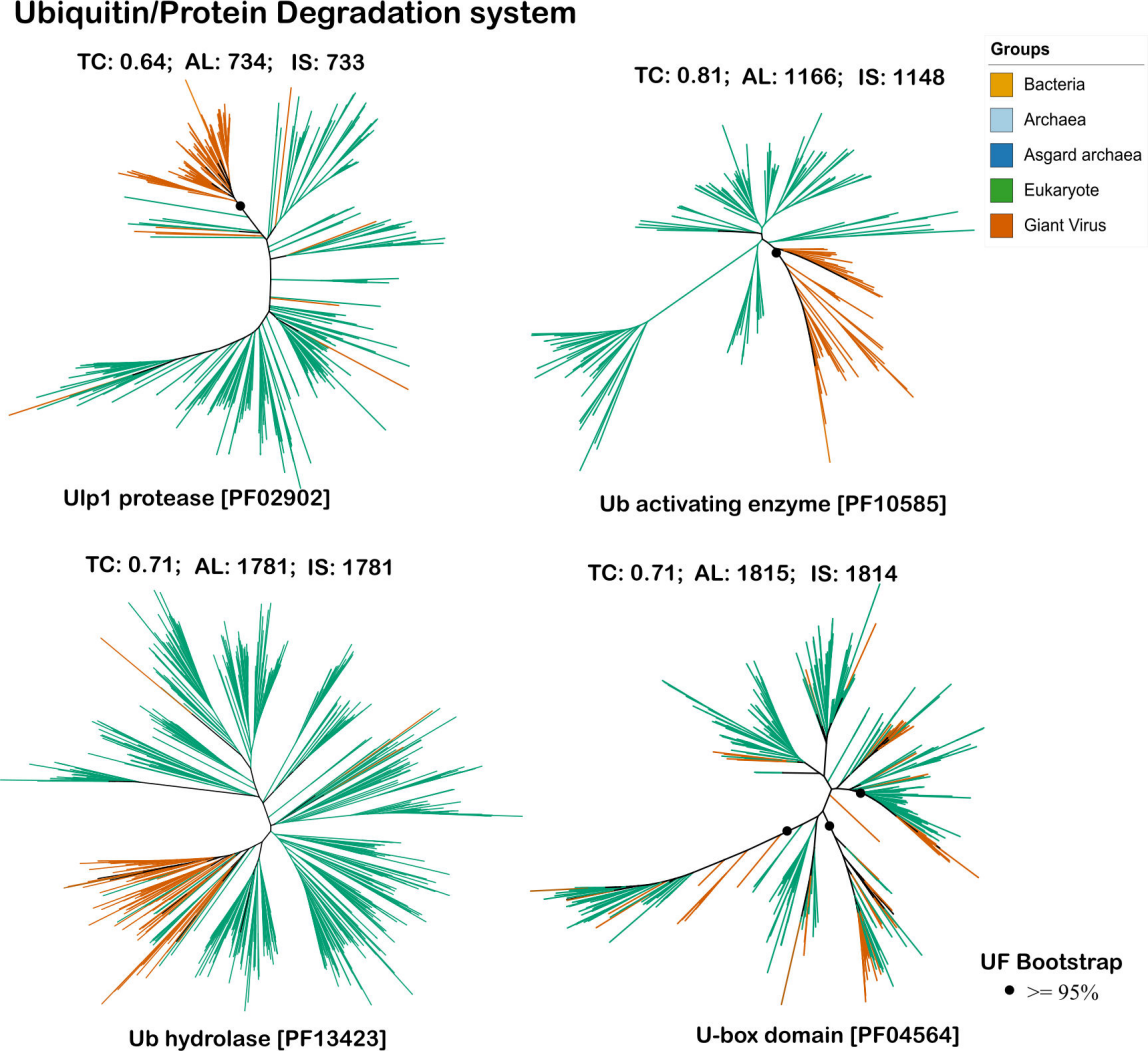
Phylogenetic trees for vESPs involved in ubiquitin-protease system. TC refers to the tree certainty value for the given tree, AL refers to alignment length, and IS refers to the number of parsimony informative sites. The dots represent ultrafast bootstrap support values greater than 95%, only for the major viral branches. All bootstrap support values are provided in supplementary figures, Fig. S6.

Our phylogenetic analysis provides insights into the acquisition and evolution of ubiquitin system proteins in nucleocytoviruses. Notably, we observed larger viral clades for Ulp1 protease, Ub-activating enzymes, and Ub hydrolases, while distinct smaller clades indicative of multiple independent acquisitions were evident for the U-box domain protein. Among these, the UBA vESPs were relatively deeply-rooted, indicating older gene exchange events compared to other ubiquitin system proteins (Fig. 6; Fig. S3).

SUMO (small ubiquitin-like modifier) proteases, such as the Ulp1 protease, are integral in the removal of SUMO proteins from target substrates. This removal is crucial for regulating various cellular functions, including protein localization, stability, and activity (65). Past research has shown that ASFV (member of *Asfuvirales*) encodes a protease belonging to the SUMO-1-specific protease family (66). This protease is essential for the maturation of ASFV particles, facilitating the cleavage of polyprotein precursors into functional viral proteins required for virus assembly and infectivity (67, 68). In our analysis, we identified Ulp1-like proteases within the *Asfuvirales*, which clustered at the base of the *Megaviricetes* with an ultrafast bootstrap support value of 95% (Fig. 6). The presence of Ulp1-like proteases in a wide range of nucleocytovirus lineages suggests that this protein plays a key role in the infection of diverse eukaryotic hosts. Previous research has reported the presence of a U-box domain in the brown tide virus Aureococcus anophagefferens virus (AaV) (69), while another study suggested that this U-box domain might be present and function in E3 ubiquitin ligase (70). These findings indicate that the U-box domain in giant viruses may play a role in ubiquitination processes, potentially influencing viral protein regulation and function. Similarly, experimental studies have demonstrated the functional relevance of ubiquitin signaling proteins in various nucleocytoviruses that infect animals. In ASFV and poxviruses, the ubiquitin-proteasome complex is essential for viral gene expression, virus factory formation, and DNA replication, with proteasome inhibition disrupting these processes (33, 71). Additionally, the role of E2 Ub-conjugating enzyme in ASFV infection (33) and ubiquitin E3 ligases in poxvirus virulence has also been described (72). These findings underscore the complex evolutionary strategies of nucleocytoviruses in acquiring and repurposing ubiquitin system proteins to enhance their replication and pathogenicity.

### Quantitative comparison of tree depth across vESP categories

**Fig 7:**
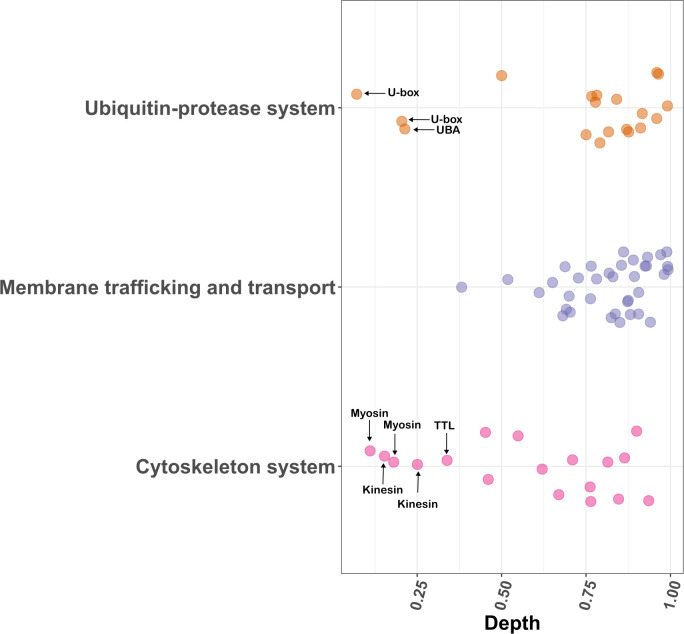
Phylogenetic tree depth for membrane trafficking and transport proteins, ubiquitin, and cytoskeleton system. *X*-axis value 0 indicates root while 1 indicates tip in the phylogenetic tree. Each dot represents one viral clade (a phylogenetic tree can have one or multiple clades).

In order to compare the relative timing at which different protein families emerged in the *Nucleocytoviricota*, we examined ultrametric trees of all protein families analyzed in this study and identified the depth at which the first appeared in this viral phylum. Because these proteins are often found in only eukaryotes and viruses and there are no suitable outgroups for rooting the trees, we relied on midpoint rooting to provide an estimate of the root position. Scenarios in which the most basal-branching lineage was viral were given a depth of 0 and most terminal-branching lineage were given a depth of 1 (Fig. 7). Although the evolutionary rates of these proteins vary, this comparison may nonetheless provide a general framework for assessing the relative timing of different transfer events. Proteins involved in vesicular trafficking and transport mostly had a depth score of greater than 0.5, suggestive of more recent acquisition. Though few vESPs for the ubiquitin system (few U-box viral clades and ubiquitin-activating enzyme) had a depth score of <0.5, most of them had a depth greater than 0.5, revealing comparative recent acquisitions. For cytoskeletal structure, the depth of vESP clades was widely distributed, revealing that viral-encoded cytoskeleton structure proteins are the product of both older and more recent acquisitions (Fig. 7). For example, myosin, tyrosine tubulin ligase, and one kinesin viral clade had a tree depth of <0.3, while the actin and dynamin tree depth scores were >0.5. The relatively shallow depth of the viral actin clade contrasts sharply with a recent report that suggested that viral actins are potentially the precursors of eukaryotic actin (31). Viral actins are relatively rare in nucleocytovirus genomes, and we detected them only in genomes in the order *Imitervirales*, and it is therefore challenging to reconstruct their evolutionary history. It is possible that viral actins are the result of more recent viral acquisitions, as our analyses suggest, but it is also possible our approach of using midpoint rooting trees provides inaccurate dates for viral clades with only a small number of representatives.

## DISCUSSION

In this study, we present a phylogenetic analysis of ESPs encoded in nucleocytoviruses to evaluate virus-host gene transfers that have occurred throughout the co-evolution of eukaryotes and their viruses. Phylogenetic analysis of vESPs involved in ubiquitin signaling, vesicular trafficking, and cytoskeletal structure revealed topologies in which vESPs were nested within broader clades of eukaryotic proteins (Fig. 4 to 6), indicative of eukaryote-to-virus gene transfer events. In most cases, it is not possible to root these trees given the lack of homologs in other cellular domains, but the placement of almost all vESPs within multiple distinct lineages nested within eukaryotic clades is a strong evidence that viruses acquired these vESPs after the establishment of the main eukaryotic supergroups. Moreover, some protein families exhibited multiple distinct vESP clades, suggesting that viruses acquired these proteins multiple times independently throughout their evolution. Our results also show that the depth of the vESP clades varied between trees. For example, whereas most membrane trafficking and transport vESPs are scattered broadly among eukaryotic homologs (Fig. 5), the cytoskeleton vESPs and ubiquitin system vESPs form some large, deep clades in midpoint-rooted trees (Fig. 4 and 6). Collectively, this suggests that the vESPs were acquired by viruses over a range of different timescales, with shallow vESP clades representing recent acquisitions and deeper clades representing older gene transfer events. Our finding of broad timescales over which viral genes were acquired is largely consistent with previous studies that have focused on viral genes from other functional categories, such as translation (16, 40, 73).

The timing and number of independent viral gene acquisitions provides insight into the functional roles of these genes in viral infection cycles. Older acquisitions, such as those observed for certain cytoskeletal and ubiquitin system proteins, potentially represent viral proteins with conserved roles in infection that are critical across a wide range of virus-host pairs. These proteins enable viruses to effectively manipulate host cellular processes, ensuring successful infection and replication over evolutionary timescales. For example, Ulp1 protease, involved in processing SUMO modifications, appears to have been co-opted for enhancing viral protein maturation and assembly, highlighting its importance in maintaining viral fitness (66). Similarly, the presence of TTL, which plays a role in tubulin modification and stabilization (51), suggests its acquisition may contribute to viral strategies for manipulating host cell cytoskeletons to facilitate viral replication and movement. TTL’s role in modulating microtubule dynamics could be critical for viral transport within the host cell, further underscoring the significance of functional integration of host-derived components into the viral lifecycle. In contrast, more recent acquisitions, such as membrane trafficking proteins, including synaptobrevin and mitochondrial carrier proteins, may reflect more host-specific adaptations. The scattering of these proteins among various eukaryotic homologs indicates that viruses continuously integrate new components to adapt their replication strategies in response to changing host environments and evolving defense mechanisms. Recent acquisitions potentially enable viruses to exploit host cellular mechanisms in a manner more specific to the particular host lineage they infect.

Overall, our study highlights the dynamic nature of giant virus evolution and adaptation through the acquisition of host-derived ubiquitin systems, cytoskeletal proteins, and membrane trafficking proteins. These evolutionary strategies may enable viruses to optimize their replication processes and evade host immune responses. Broadening our understanding of these gene acquisition patterns provides insights into viral adaptation mechanisms and virus-host interactions.

## MATERIALS AND METHODS

### Compilation of genomic data sets used in this study

Prior to phylogenetic analysis, we compiled a set of eukaryotic, archaeal, bacterial, and nucleocytovirus genomes. We downloaded high-quality eukaryotic genomes from the eggNOG v.5.0 database. Because eggNOG has relatively few protist genomes, we also included complete and chromosome-level genomes for select protists available on the National Center for Biotechnology Information databases as of 8 October 2021. For bacterial and archaeal genomes, we retrieved genomes from the Genome Taxonomy Database (GTDB, v.95). To enrich our database in Asgard archaeal genomes, we also included Asgard archaea genomes as reported in reference 47 that were not already present in the GTDB. For giant viruses, we used a set of curated genomes available on the Giant Virus database (https://faylward.github.io/GVDB/) that have been described previously (1). Finally, we came up with a set of 97 archaeal, 182 Asgard archaeal, 230 bacteria, 343 nucleocytoviruses, and 127 eukaryotic genomes. For eukaryotic genomes, we used protein predictions available on eggNOG, and for all other taxa, we predicted proteins using Prodigal v.2.6.3 with default parameters (74). A full list of all genomes used is available in 10.5281/zenodo.13830164.

### ESP detection, clustering, and functional annotation

For *de novo* identification of ESPs, we searched bacterial, archaeal, viral, and eukaryotic genomes against the Pfam database and searched for domains that were enriched in eukaryotes. For this, we used the hmmsearch command in the HMMER3 v.3.3 package with the “--cut_nc” parameter (75), with the Pfam database version 32.0 (76) as a query. To determine which Pfams were ESPs, we first removed all Pfams that were not present in >95% of all eukaryotic genomes, leaving us with a set of 763 protein families (see Table S1). We then sought to cluster these Pfams into groups based on their distribution across the taxonomic groups in our analysis. For this, we employed an unsupervised learning approach using self-organizing maps in R version 3.6.3, implemented with the kohonen package (script for this is available in https://github.com/sangitakarki/Self-Organising-Maps). To choose the optimal number of clusters, we used the heuristic “elbow method” and ultimately arrived at six groups (Fig. 1A). To determine the role of these eukaryotic proteins, we performed functional annotation of proteins using hmmsearch against the KEGG (Kyoto Encyclopedia of Genes and Genomes) database (KEGG release 99.0, v.2021-08-01) (77). Since the same genes had hits to multiple KEGG categories, we manually curated the results from the KEGG annotation to refine categories. The redundancy of eukaryotic protein sequences made it challenging for future analysis; we therefore downsampled the sequences using the seqkit sample function (78). We also used cd-hit version 4.8.1 with default parameters (79) to dereplicate nearly identical protein sequences. Extreme short sequences were also removed from the overall protein sequences so as to avoid any phylogenetic signal interference.

### Phylogenetic analysis

For phylogenetic reconstruction, we retrieved protein sequences with hits to the Pfams under investigation, aligned them together using Muscle5 (parameters “-super5” for input sequences), and used trimAl v.1.4. rev15 for alignment trimming (parameter -gt 0.1) (80). We then reconstructed maximum likelihood phylogenetic trees using IQtree v.2.1.2 (81) with the option -bb 1000 to generate 1,000 ultrafast bootstraps (82), -m MFP to determine the best-fit model (several best-fit substitution models for different trees were selected based on Bayesian Information Criterion) (83) , -nt AUTO and --runs 5 to select the highest likelihood tree. Most protein trees were inferred under the LG (84) and VT (85) amino-acid exchange rate matrices, amino-acid frequencies (F), and variation in evolutionary rates across sites (R) (86), with different numbers of categories.

All trees were visualized using the Interactive Tree of Life (87). If we observed long branches that potentially represented rogue taxa, we removed these sequences from the analysis and re-performed alignment, trimming, and phylogenetic reconstruction. All trees can be accessed here: https://itol.embl.de/shared/tsSK9OEY2ezD and all alignments can be found here: 10.5281/zenodo.13830164.

### Assessing tree quality and tree depth

In order to evaluate the phylogenetic congruency of the trees, we used the TC metric, which provides a measure of overall tree quality (88). This was done in RaxML version 8.2.12 (89) (options -t and -z used to input the tree file and bootstrap file, respectively). All TC scores are available in Table S2 as well as in the main text figures. We calculated the alignment length and number of parsimony informative sites using phyKIT v.2.0.1 (90). We calculated the depth of viral clades in trees using a script that utilizes ape package (91) in R, bio-python, and python. All associated scripts can be found in https://github.com/sangitakarki.

## SUPPLEMENTAL MATERIAL

Supplemental figures jvi.01715-24-s0001.docx Figures S1 to S6.

Table S1 jvi.01715-24-s0002.xlsx Full list of proteins and associated Pfam generated in this study.

Table S2 jvi.01715-24-s0003.xlsx Description of cytoskeleton, ubiquitin, and membrane trafficking and transport proteins used in this study.

Table S3 jvi.01715-24-s0004.xlsx Percentage sequence identities between viral and host proteins.

ASM does not own the copyrights to Supplemental Material that may be linked to, or accessed through, an article. The authors have granted ASM a non-exclusive, world-wide license to publish the Supplemental Material files. Please contact the corresponding author directly for reuse.
