# Supplementary material for: Evolution of ubiquitin, cytoskeleton, and vesicular trafficking machinery in giant viruses
Source: J Virol. 2025 Feb 11;99(3):e01715-24. doi: 10.1128/jvi.01715-24 (PMC11915834; doi:10.1128/jvi.01715-24)
Supplement: Supplemental figures — Figures S1 to S6. [file jvi.01715-24-s0001.docx]

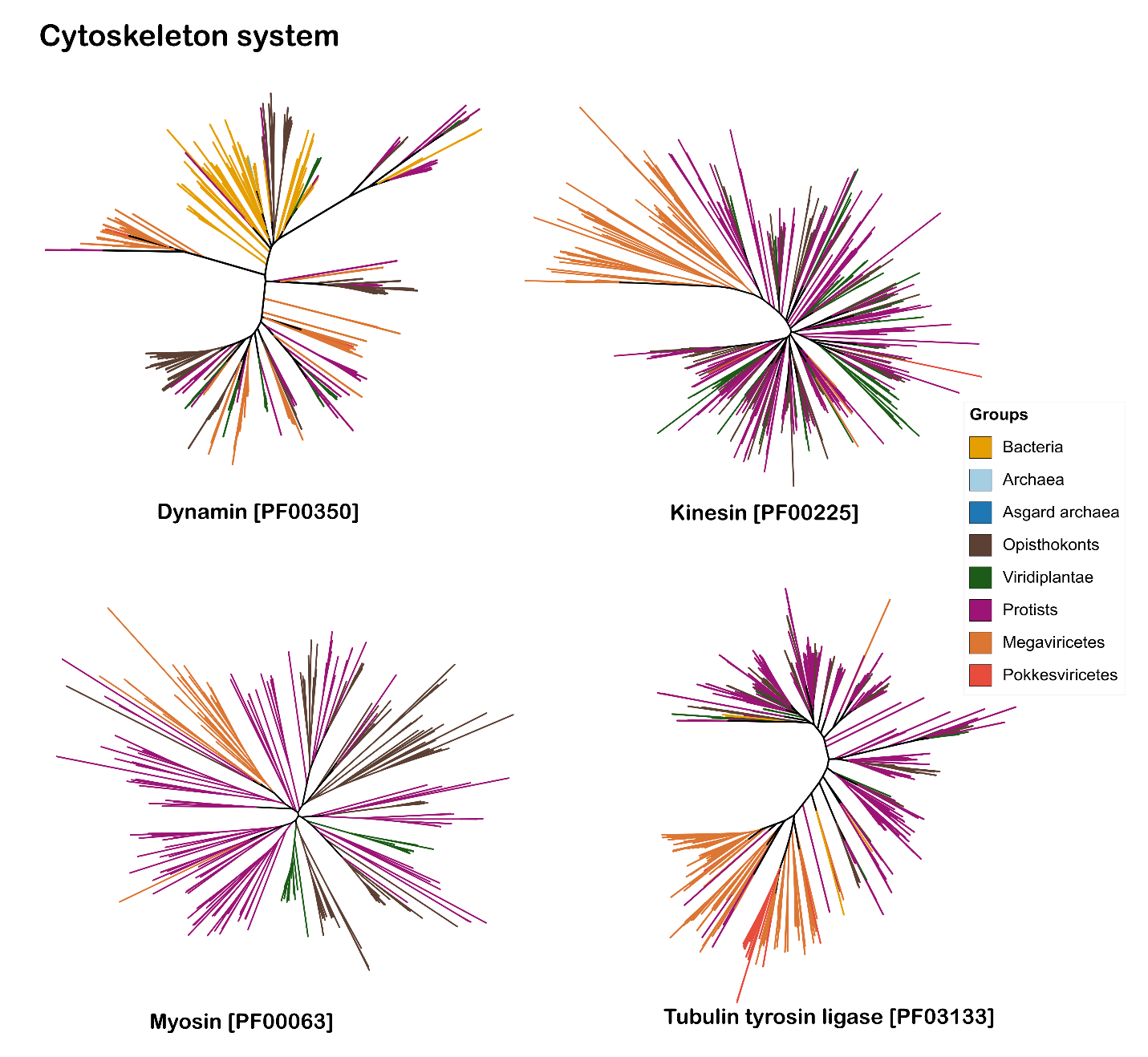


Fig S1. Phylogenetic tree for cytoskeletal structure proteins. The tree shows different groups of eukaryotes and giant viruses


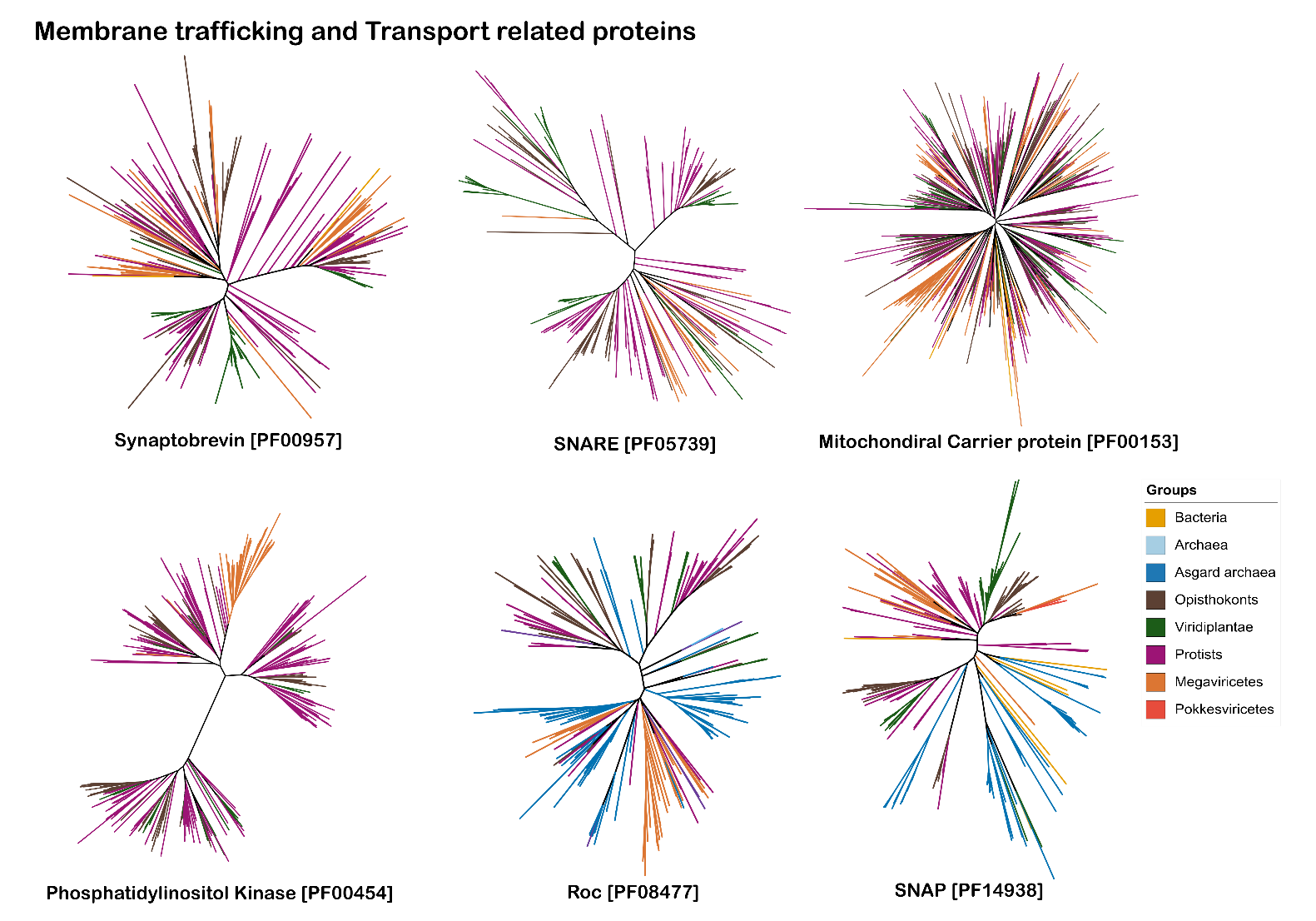


Fig S2. Phylogenetic tree for membrane trafficking and transport proteins. The tree shows different groups of eukaryotes and giant viruses


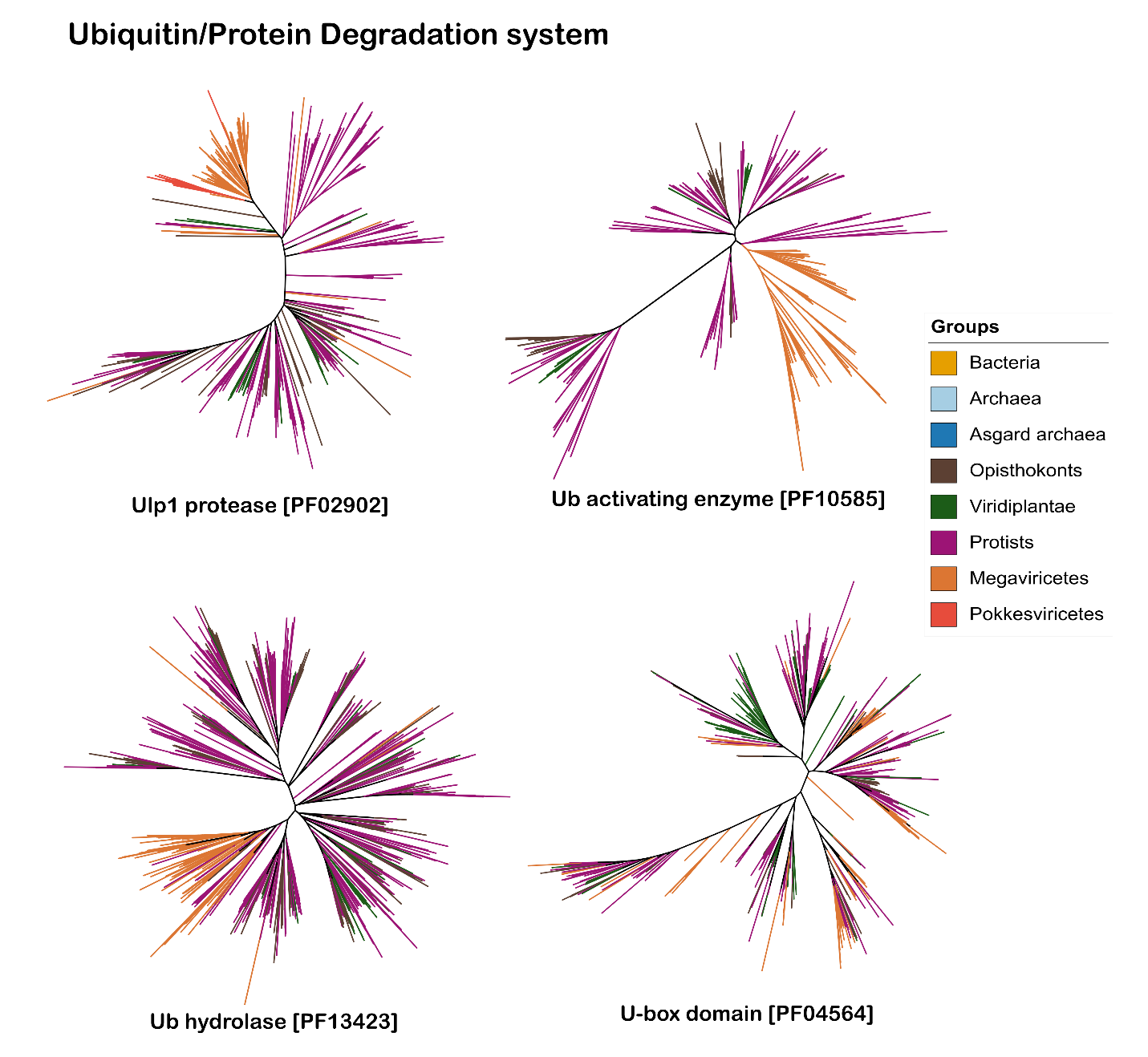


Fig S3. Phylogenetic tree for ubiquitin system proteins. The tree shows different groups of eukaryotes and giant viruses


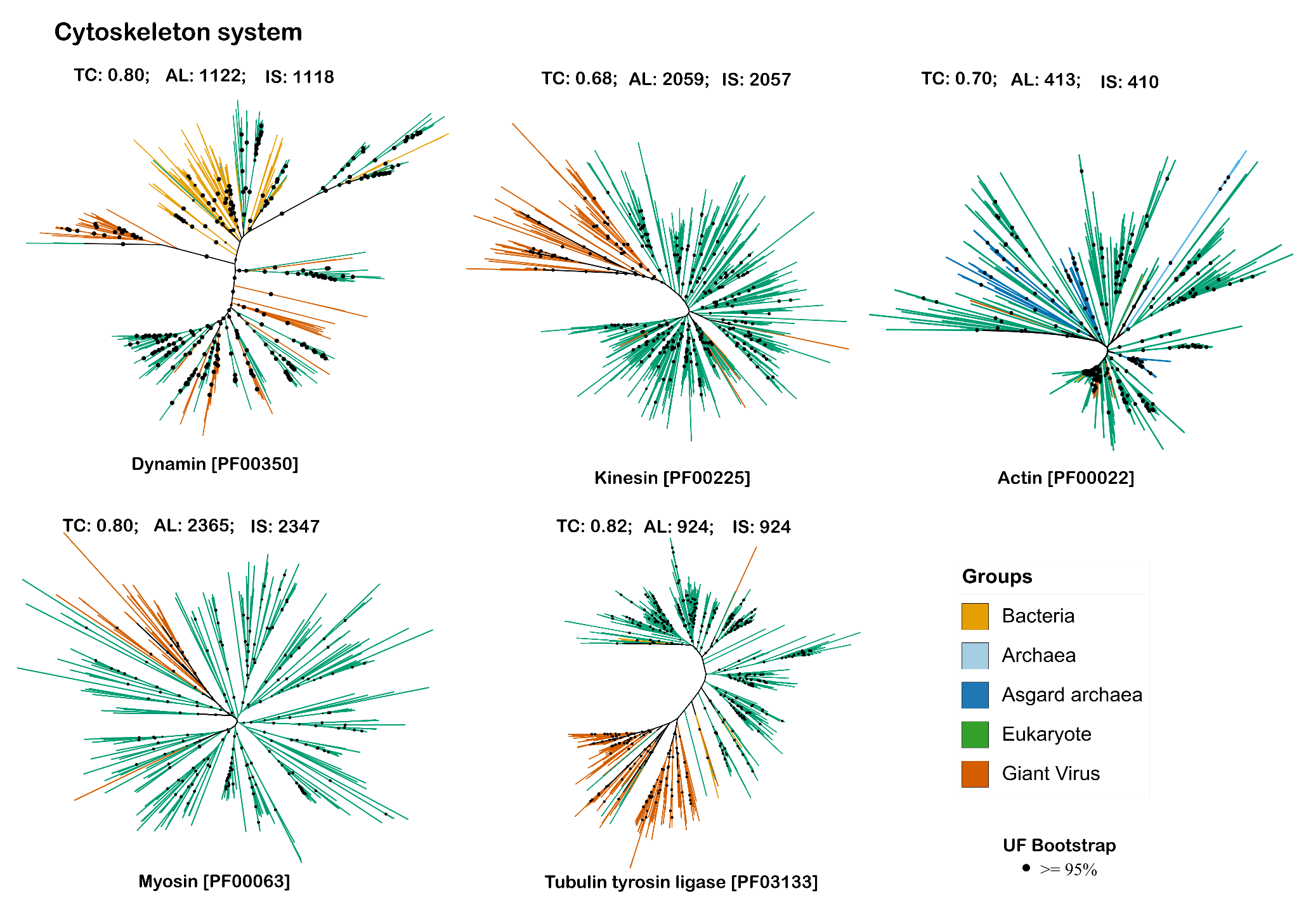


Fig S4. Phylogenetic tree for cytoskeletal structure proteins. The black dot represents UF bootstraps greater than 95%


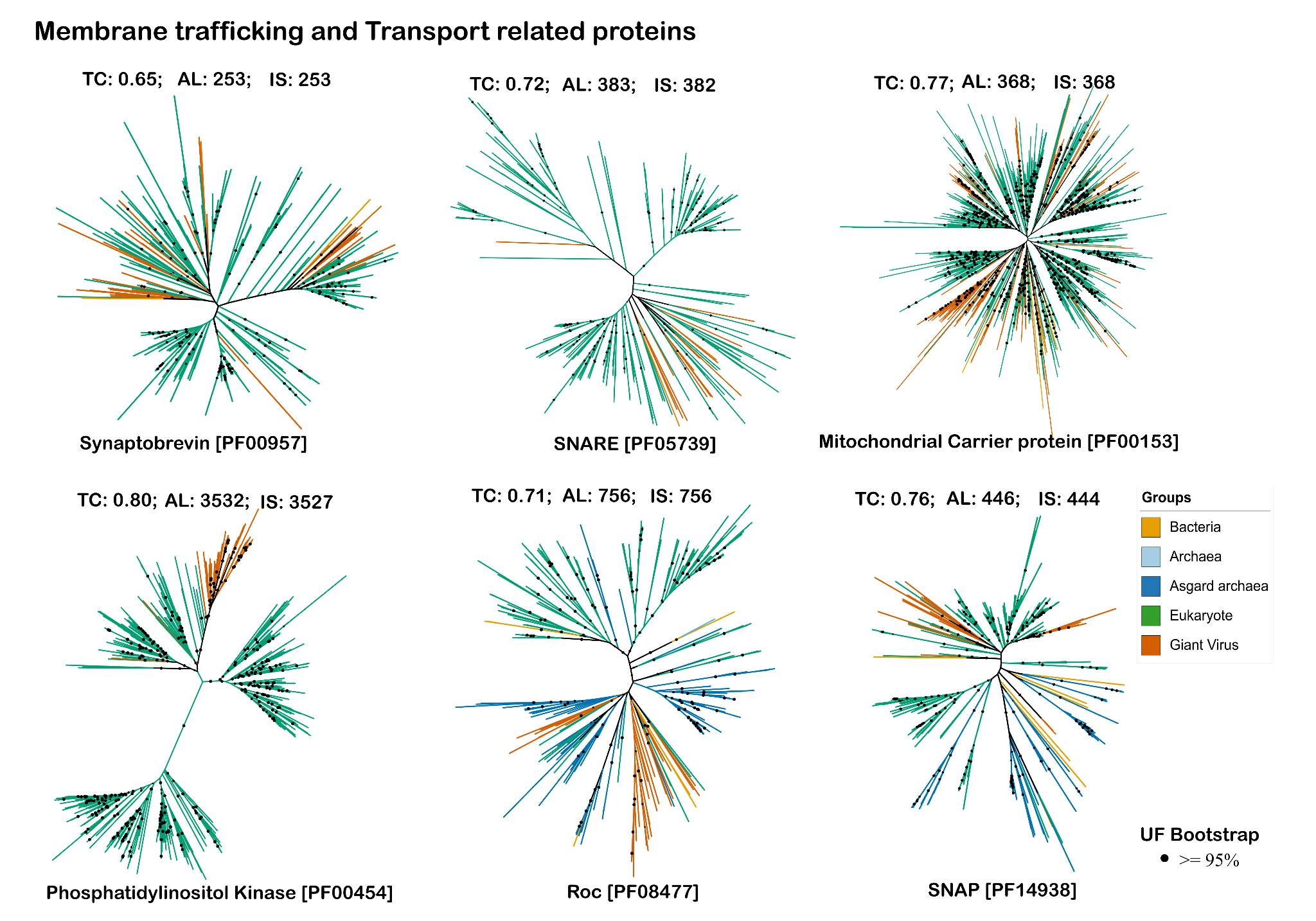


Fig S5. Phylogenetic tree for membrane trafficking and transport proteins The black dot represents UF bootstraps greater than 95%


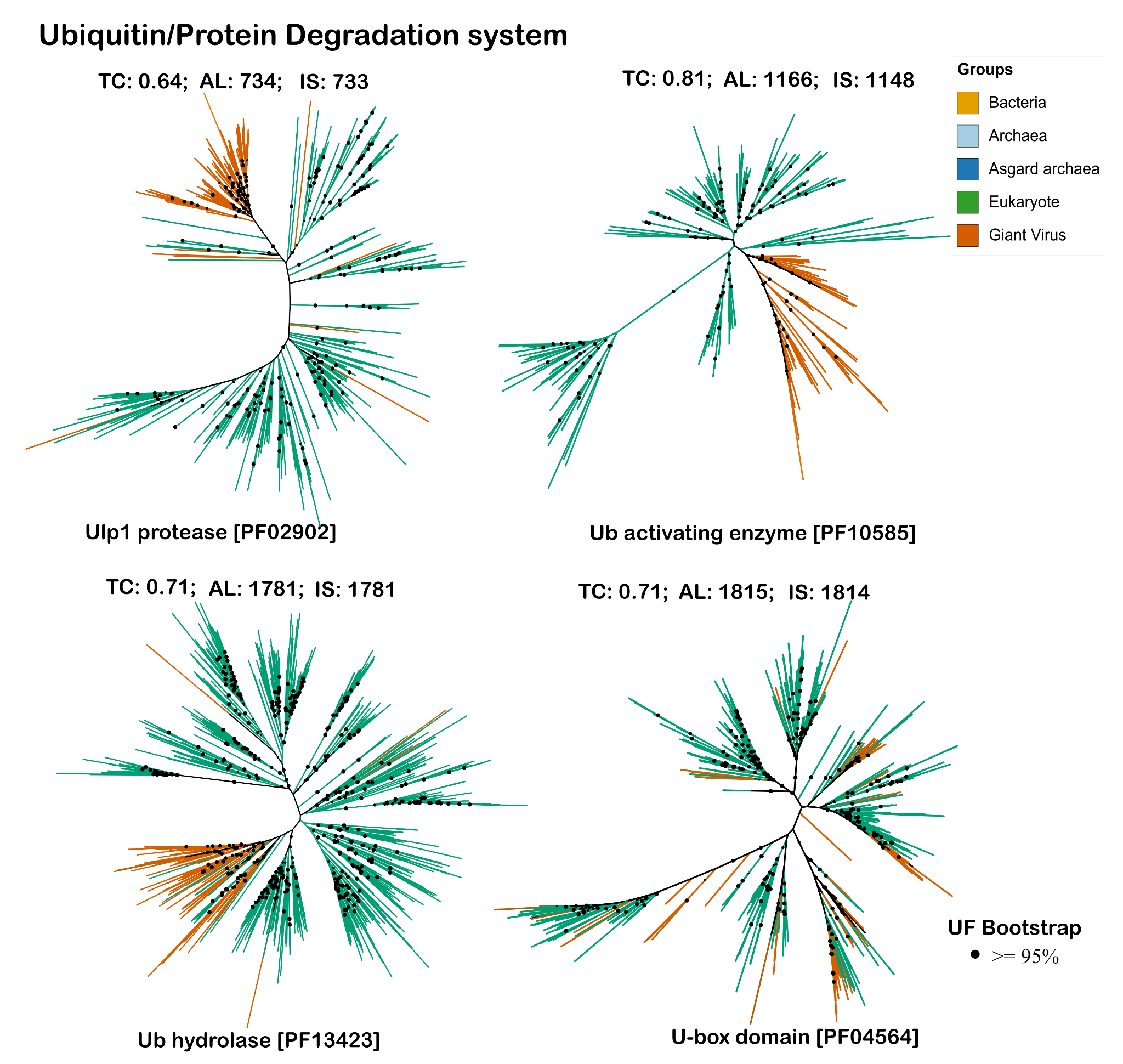


Fig S6. Phylogenetic tree for ubiquitin system proteins. The black dot represents UF bootstraps greater than 95%
